# Integrated Crop Management Practices Improve Grain Yield and Resource Use Efficiency of Super Hybrid Rice

**DOI:** 10.3389/fpls.2022.851562

**Published:** 2022-03-30

**Authors:** Jun Deng, Matthew Tom Harrison, Ke Liu, Jiayu Ye, Xin Xiong, Shah Fahad, Liying Huang, Xiaohai Tian, Yunbo Zhang

**Affiliations:** ^1^Hubei Collaborative Innovation Center for Grain Industry, Agriculture College, Yangtze University, Jingzhou, China; ^2^Tasmanian Institute of Agriculture, University of Tasmania, Burnie, TAS, Australia; ^3^Hainan Key Laboratory for Sustainable Utilization of Tropical Bioresource, College of Tropical Crops, Hainan University, Haikou, China; ^4^Department of Agronomy, The University of Haripur, Haripur, Pakistan

**Keywords:** super hybrid rice, management, dry matter, grain yield, nitrogen use efficiency, radiation use efficiency

## Abstract

Super hybrid rice genotypes have transformed the rate of genetic yield gain primarily due to intersubspecific heterosis, although the physiological basis underpinning this yield transformation has not been well quantified. We assessed the radiation use efficiency (RUE) and nitrogen use efficiency (NUE) of novel hybrid rice genotypes under four management practices representative of rice cropping systems in China. Y-liangyou 900 (YLY900), a new super hybrid rice widely adopted in China, was examined in field experiments conducted in Jingzhou and Suizhou, Hubei Province, China, from 2017 to 2020. Four management practices were conducted: nil fertilizer (CK), conventional farmer practice (FP), optimized cultivation with reduced nitrogen (OPT_–N_), and optimized cultivation with increased nitrogen (OPT_+N_). Yield differences across the treatment regimens were significant (*p* < 0.05). Grain yield of OPT_+N_ in Jingzhou and Suizhou were 11 and 12 t ha^–1^, which was 14 and 27% greater than yields obtained under OPT_–N_ and FP, respectively. Relative to OPT_–N_ and FP, OPT_+N_ had greater panicle numbers (9 and 18%), spikelets per panicle (7 and 12%), spikelets per unit area (17 and 32%), and total dry weight (9 and 19%). The average RUE of OPT_+N_ was 2.7 g MJ^–1^, which was 5 and 9% greater than that of OPT_–N_ and FP, respectively, due to higher intercepted photosynthetically active radiation (IPAR). The agronomic efficiency of applied N (AE_N_) of OPT_+N_ was 17 kg grain kg^–1^ N, which was 9 and 68% higher than that of OPT_–N_ and FP. These results show that close correlations exist between yield and both the panicles number (*R*^2^ = 0.91) and spikelets per panicle (*R*^2^ = 0.83) in OPT_+N_. We conclude that grain yields of OPT_+N_ were associated with greater IPAR, RUE, and total dry matter. We suggest that integrated cropping systems management practices are conducive to higher grain yield and resource use efficiency through expansion of sink potential in super hybrid rice production.

## Introduction

Rice represents a staple food for nearly two-thirds of the Chinese population (Fahad et al., 2021). To match the rising dietary needs of China in the 21st century, total grain production must rise to at least 7 × 10^5^ t per year (Zhu et al., 2010). Assuming that total global arable productive land used for rice production remains unchanged (Faridvand et al., 2021; Raza et al., 2021; Rezaei-Chiyaneh et al., 2021a,b), intensification of rice yields must be achieved to ensure food security. Such intensification must occur in sustainable, profitable, and socially-acceptable ways, without degrading natural resources, causing excessive nutrient losses or eutrophication, loss of biodiversity, or increasing greenhouse gas emissions (Alcock et al., 2015; Liu et al., 2020a; Harrison et al., 2021). It is also likely that climate change will incite more frequent extreme events such as droughts and flash flooding, which in many regions will seriously challenge the consistency of agricultural productivity from year to year (Harrison et al., 2011, 2012a,b; Phelan et al., 2015). While the climate crisis will have global implications (Harrison, 2021), the Arctic, Africa, Australia, small islands, and Asian megadeltas are likely to be among the most severely impacted by increasing frequencies of extreme climatic events.

In 1996, a program for breeding super hybrid rice was established by the Ministry of Agriculture and Rural Affairs of the People’s Republic of China to meet the burgeoning food demand of the nation (Yuan, 2018). By 2019, 132 rice genotypes had met the criteria of super rice cultivars; they were defined as elite cultivars with high yield potential, larger sink capacity, higher photosynthetic rate, stronger and more vigorous root systems, and greater dry matter production (Ai et al., 2011). Yield gain over the time was clear: in 2000, the cultivar Liangyoupeijiu yielded 10.6 t ha**^–^**^1^; in 2004, Y-liangyou 1 attained 12.1 t ha**^–^**^1^; in 2011, Y-liangyou two yielded 13.9 t ha**^–^**^1^; in 2014, Y-liangyou 900 reached 15.4 t ha**^–^**^1^; and in 2018, Xiangliangyou 900 smashed the world record, reaching a yield of 17 t ha**^–^**^1^ (Ma and Yuan, 2015; Yuan, 2017). China’s research and development of rice with superior yields have won global accolades. However, continual genetic yield gains and improvement of resource use efficiency are paramount, if the demand of the growing global population is to be sustained. While much research has been done on individual agronomic interventions (e.g. effects of fertilizer treatments on yields), there is much less systems research that integrates and assesses the benefits of multiple management practices across multiple metrics (e.g. Harrison et al., 2016). We address this gap in the present paper.

At the time of writing, the rice cropping area in China accounts for 20% of the world’s rice plantation, while associated N fertilizer usage accounts for 37% of global rice N use (Liu et al., 2009). Average N application rates in Chinese rice cropping systems of 180 kg ha^–1^ are 75% higher than fertilization rates used elsewhere (Zhou et al., 2010). To achieve higher yields, farmers generally adopt a “more fertilizer and much water” management practice, in which more nitrogen fertilizer is often applied in the early growth stages, while the importance of N for the later crop development stages was largely ignored. Supraoptimal N fertilizer application during the early crop life cycle not only inhibits potential yield and nitrogen use efficiency (NUE) but also increases the risk of water waste and environmental pollution (Adeyemi et al., 2020; Diatta et al., 2020). Excessive N fertilizer application may result in N losses through many avenues, including N leaching, runoff in surface water, and potent greenhouse gas emissions, including nitrous oxide (Rawnsley et al., 2019; Christie et al., 2020). When applied in early crop development, excessive N fertilizer stimulates tillering, resulting in dense rice canopies with reduced ventilation and light penetration and, thus, reduced solar radiation interception at lower canopy layers and increased incidence of pest and disease (Zhang and Gong, 2014; Liu et al., 2019a). The culmination of these factors tends to severely constrain potential yields (Harrison et al., 2014a). Conducting late-season N fertilization maintains the photosynthetic capacity of younger rice leaves prolongs green area duration, improves solar radiation interception, and increases dry matter accumulation, these factors typically lead to greater rice yields (Fu et al., 2019).

Plant density is a key management tool enabling manipulation of crop canopies, physiological activity, fertilizer uptake and use, weed control, and crop yield (Harrison et al., 2012a,b). Planting density may also influence phenology in some contexts (Ibrahim et al., 2018). In recent years, super hybrid rice cultivation has gravitated toward lower plant populations to enable greater yields (Yuan, 2017). Although rice crops with lower plant density are beneficial to individual plant growth, in some contexts, this may lead to insufficient panicle numbers and lower yields (Yang et al., 2009). Relatively dense planting can optimize the population structure, increase radiation interception and panicle numbers, leading to greater dry matter, thus improving yield. Dense plant densities can also improve light interception in the early stage of rice, increasing water use efficiency, light interception, and N fertilizer use efficiency (Ibrahim et al., 2019; Liu et al., 2020b).

With increasing water scarcity, contemporary research has transitioned toward the investigation of systematic water-saving management options for rice, such as alternating wet and dry and intermittent irrigation (Pan et al., 2017; Liu et al., 2021). These techniques have been shown to have favorable effects on rice growth and development as well as yield formation. Therefore, optimization of management by the interaction of environment and genotype increases the likelihood of reaching yield potential and maximum resource use efficiency (Harrison et al., 2017; Ibrahim et al., 2019; Liu et al., 2020c).

To improve the resource use efficiency of rice, previous research has investigated integrated management techniques of high-yielding genotypes, including effects of cultivation (Liu et al., 2009; Harrison et al., 2014b; Shah et al., 2017a,b). For example, N management techniques combining N fertilizer application limit soil fertility and precision and field nutrient management techniques have been examined (Liu et al., 2009; Shah et al., 2021). Soil water management, such as alternating wet and dry, intermittent wetting, and controlled irrigation, can significantly improve water use efficiency while promoting rice growth, rice development, and yield formation (Harrison et al., 2014b; Liu et al., 2020c). Variation in phenology is also important since crop development determines the life cycle duration, flowering time, and growing season duration (Liu et al., 2020d).

As for most agronomic experiments, field experiments tend to be limited to strategically planned management events that, once passed, are cemented in time and can no longer be changed. Outcomes from such strategic decisions have ensuing implications for growth for the remainder of the crop life cycle (Harrison et al., 2012a,b). For example, although increasing N fertilizer improves rice yield within a certain range, the efficiency of N fertilizer decreases above a certain threshold of N fertilization. Similarly, increasing plant density can improve panicle number, but excessive planting densities stifle light interception and encourage pest and disease infection, as mentioned above. Moreover, excessive or deficient water availability affects plant growth and development and, potentially, rice yield (Peng et al., 2002; Zhou et al., 2010). These insights suggest that holistic, integrated, and strategic consideration of multifaceted management factors such as N fertilizer, transplanting density, and soil moisture are conducive to greater resource use efficiency and yield potential of modern rice hybrids: the highest yields can only be attained with careful and deliberate programming of crop management throughout the crop life cycle.

To improve yields through resource-use efficiency gains, here we pioneer and test novel and integrated cropping systems practices. To address this aim, we conducted a field experiment using Y-liangyou 900 (YLY900), the representative high-yield super hybrid rice variety in Jingzhou and Suizhou, China, from 2017 to 2020. We aimed to (1) compare differences in radiation use efficiency (RUE), nitrogen use efficiency (NUE), growth, and grain yield of super hybrid rice under four representative management systems and (2) elicit relationships between NUE and RUE with dry matter accumulation of grain yield in super hybrid rice to better understand the reasons of high yield and resource use efficiency of super hybrid rice.

## Materials and Methods

### Study Site

Field experiments were conducted at the experimental farm of Yangtze University (112°31′E, 30°21′N) in Jingzhou from 2017 to 2020 and in Suizhou (113°35′E, 28°39′N) from 2017 to 2019, Hubei Province, China. Daily maximum temperatures were 38, 38, 38, and 37°C and the daily minimum temperatures were 16, 15, 18, and 16°C. Total cumulative solar radiation during crop growth duration was 1,911, 2,124, 2,168, and 1,807 MJ m^–2^ in 2017, 2018, 2019, and 2020 in Jingzhou (Supplementary Figure 1), respectively. Daily maximum temperature, daily minimum temperature, and total solar radiation during the rice growth duration in 2017 were 38, 15°C, and 2,025 MJ m^–2^; in 2018, were 38, 12°C, and 2,221 MJ m^–2^; and in 2019, 39, 13°C, and 2,102 MJ m^–2^ in Suizhou, respectively (Supplementary Figure 2). The average maximum temperature in Suizhou was 0.8–1.4°C higher, while the minimum temperature was 2.4–3.4°C lower than that in Jingzhou. Total solar radiation in Suizhou was 7% higher than that in Jingzhou.

### Test Material

Y-liangyou 900, super hybrid rice with Y58S (♀) and R900 (♂), was used in this study. YLY900 is widely planted in southern China and is recommended by the China National Hybrid Rice Research and Development Center in China.

### Experimental Design and Site Details

Soil samples were taken from the upper 20 cm of the soil, with soil property data averaged across the 4 years. The soil in Jingzhou was a calcareous alluvial having pH 6.8, 18.5 g kg**^–^**^1^ organic matter, 110.5 mg kg**^–^**^1^ alkali-hydrolyzable N, 25.0 mg kg**^–^**^1^ available P, and 105.5 mg kg**^–^**^1^ available K. The soil in Suizhou was clay with pH 6.52, 20.3 g kg**^–^**^1^ organic matter, 135 mg kg**^–^**^1^ alkali-hydrolyzable N, 27.2 mg kg**^–^**^1^ available P, and 145.5 mg kg**^–^**^1^ available K.

Pregerminated seeds were sown in a seedbed. Seedlings were transplanted between 28 and 32 days after planting to field plots with two seedlings per hill. The transplantation dates were June 3, 2017, June 1, 2018, June 10, 2019, and June 10, 2020, in Jingzhou; the transplantation dates were May 20, 2017, May 28, 2018, and May 29, 2019, in Suizhou.

Experiments were conducted for four continuous years in the two sites using four treatment regimens (Table 1). The total N applied in the four treatments in Jingzhou was 0 kg N ha^–1^ [no fertilizer (CK)], 210 kg N ha^–1^ [conventional farmer practice (FP)], 195 kg N ha^–1^ [optimized cultivation with reduced nitrogen compared to FP (OPT_–N_)], and 270 kg N ha^–1^ [optimized cultivation with increased nitrogen compared to FP (OPT_+N_)]; in Suizhou, we applied 0 kg N ha^–1^ (CK), 250 kg N ha^–1^ (FP), 210 kg N ha^–1^ (OPT_–N_), and 270 kg N ha^–1^ (OPT_+N_). Urea, potassium chloride, as well as superphosphate and zinc sulfate, were also used. Fertilizer N applied relative to phenology was also varied for each treatment: for FP, 70% was applied a basal treatment and 30% at tillering; under OPT_–N_, 50% was applied as a basal treatment, 20% at tillering, and 30% at panicle initiation (PI); under OPT_+N_, N application at basic, midtillering, PI, and topdressing phases were 50, 20, 20, and 10%, respectively. Application of phosphate fertilizer for all the treatments was applied in one time as a basal treatment and the potassium fertilizer was applied in one time under FP, two applications of 50% each for a basal treatment, and PI under OPT_–N_ and OPT_+N_. Additional treatments are shown in Table 1.

**TABLE 1:** Treatments imposed in 2017, 2018, 2019, and 2020 at the sites of Jingzhou and Suizhou.

Site	Treatment	Fertilizer	N splits (kg N ha^–1^)	Spacing (cm)	Water management
		N–P–K–Zn–Si	(B-MT-PI-FL)		
JZ	CK	0–0–0–0–0		30 × 18	Continuous flooding; shallow wetting and drying (SWD)
	FP	210–100–220–0	147–63–0–0	30 × 18	Continuous flooding; field drained after flowering
	OPT_–N_	195–100–180–5–0	97.5–39–58.5	30 × 16	Continuous flooding; SWD; Dehydrate 1 week before harvest
	OPT_+N_	270–120–240–5–150	135–54–54–27	30 × 16	Continuous flooding; SWD; Dehydrate 1 week before harvest
SZ	CK	0–0–0–0–0		20 × 20	Continuous flooding; SWD
	FP	250–125–250	175–75–0–0	20 × 20	Continuous flooding; field drained after flowering
	OPT_–N_	210–105–210–5–0	105–42–63–0	20 × 16.7	Continuous flooding; SWD; Dehydrate 1 week before harvest
	OPT_+N_	270–135–270–5–150	135–54–54–27	20 × 16.7	Continuous flooding; SWD; Dehydrate 1 week before harvest

*B, basic fertilizer; MT, midtillering; PI, panicle initiation; FL, flowering.*

All the plots were flooded for 5 days after transplanting and continuously submerged with shallow water until 5 days before PI. Plots were then drained for 5 days, irrigated at PI, and continuously flooded thereafter until the start of flowering. Then, the combined shallow water depth with wetting and drying (SWD) approach (Qing et al., 2013) was followed in all the treatments except in FP, which did not receive any irrigation after flowering. For treatments subject to SWD water management, fields were irrigated to a depth of 3.0 cm, allowed to dry, and then reirrigated to a depth of 3.0 cm before any visible cracks developed on the soil surface. Insect and disease infestation were chemically controlled throughout the crop growth cycle.

This experimental design was consistently applied across six experiments over 4 years. Daily minimum and maximum temperature and solar radiation were recorded using a Vantage Pro2 Weather Station (Davis Instruments Corporation, Hayward, CA, United States) for all the experiments across 4 years.

### Metrics Measured and Calculated

Green leaf area was measured with a leaf area meter (LI-3000, LI-COR, Lincoln, NE, United States) at flowering, and leaf area index (LAI) was calculated as the dividend of leaf area and ground area. Canopy light interception was measured between 1,100 and 1,300 at middle tillering (MT), PI, and physiological maturity (MA) using a SunScan Canopy Analysis System (Delta-T Devices Ltd., Burwell, Cambridge, United Kingdom). In each plot, light intensity at the base of the canopy was measured by placing the light bar halfway between two rows and proximal to the water surface. Three incoming light intensity readings were undertaken each within rows and between rows. Canopy light interception was calculated as the percentage of incoming light intensity that was intercepted by the canopy [100 × (incoming light intensity less light intensity inside canopy)/incoming light intensity]. Intercepted photosynthetically active radiation (IPAR) was calculated as 0.45 of total solar radiation above the canopy (Meek et al., 1984). IPAR during each growth stage was calculated using the average canopy light interception and accumulated seasonal incoming solar radiation during this growth stage [0.5 × (canopy light interception at the beginning of the growth stage + canopy light interception at the end of the growth stage)/accumulated incoming radiation during the growth stage]. IPAR across the entire growing season was calculated as the sum of IPAR during each growth stage. RUE was calculated as the ratio of total aboveground dry weight to cumulative IPAR.

Six plants were measured with three replications at flowering to determine the average tiller number. Sampled plants were divided into stem sheath, leaf, and spike before being oven dried at 105°C for 30 min and then dried at 70°C until constant weight, weighed, and ground into a powder with a grinder. Total plant tissue N content was determined using a concentrated sulfuric acid-hydrogen peroxide (H_2_SO_4_–H_2_O_2_) disinfection continuous flow analyzer. Nitrogen (N) concentrations in stems, leaves, rachi, and spikelets were determined using micro-Kjeldahl digestion, distillation, and titration (Bremmer and Mulvaney, 1982). Total N content in each plant part was calculated as the product of tissue N concentration and corresponding dry weight. The N content of all the plant parts was cumulated to obtain the total N content per plant. Nitrogen fertilizer efficiency indices were calculated as follows:


(1) $$\mathrm{Partial}\,\mathrm{factor}\,\mathrm{productivity}\,\mathrm{of}\,\mathrm{applied}\,N\,\left({\text{PFP}}_{\text{N}}\right)={\text{GY}}_{+\text{N}}/\text{FN}$$

(2) $$\mathrm{Agronomic}\,\mathrm{efficiency}\,\mathrm{of}\,\mathrm{applied}\,N\,\left({\text{AE}}_{\text{N}}\right)=\left({\text{GY}}_{+\text{N}}-{\mathrm{GY}}_{-\text{N}}\right)/\text{FN}$$

(3) $$\mathrm{Crop}\mathrm{recovery}\mathrm{efficiency}\mathrm{of}\mathrm{applied}N\left({\text{RE}}_{\text{N}}\right)\left(\%\right)=\left({\text{TN}}_{+\text{N}}-{\mathrm{TN}}_{-\text{N}}\right)\,/\,\text{FN}\times 100$$

Where TN_+N_ = total aboveground plant N accumulation in the plot that received N fertilizer; TN_–N_ = total aboveground plant N accumulation in the zero-N control; FN, N fertilizer applied; GY_+N_, grain yield in the plot that received N fertilizer; and GY_–N_, grain yield in the zero-N control.

Destructive sampling of six hills from the inner rows from each plot was carried out at midtillering (MT), PI, flowering (FL), and MA. Plant samples were separated into green leaf blades, stems (including sheath), and panicles (at flowering and MA). Separated plant parts were oven-dried at 70°C until a constant weight was obtained. The panicle number for each hill was calculated to determine the panicle number per m^2^. Panicles were hand threshed; filled spikelets were separated from unfilled spikelets by submerging in tap water. Three 30-g subsamples of filled spikelets and three 3-g subsamples of unfilled spikelets were taken to enumerate spikelet numbers. Dry weights of the rachis and spikelets were determined after oven drying at 70°C to a constant weight. Total aboveground dry weight was calculated as the total dry matter of straw, rachis, and filled and unfilled spikelets. Spikelets per panicle and grain filling percentage (100 × filled spikelet number/total spikelet number) were also calculated. Grain yield was determined from a 5-m^2^ area in each plot and standardized to a moisture content of 0.14 g H_2_O g**^–^**^1^.

### Data Analysis

Data were analyzed using ANOVA (Statistix 8, Analytical Software, Tallahassee, FL, United States); genotypic means were compared using least significant differences (LSDs) with a significance level of 0.05 unless stated otherwise.

## Results

### Grain Yield and Yield Components

Grain yields differed significantly between treatments in Jingzhou and Suizhou. Treatment yields at both the sites were ranked in OPT_+N_ > OPT_–N_ > FP > CK (Tables 2, 3). Average yields across treatments at Jingzhou of OPT_+N_, OPT_–N_, FP, and CK were 11, 10, 9, and 7 t ha^–1^, respectively. Average yields across 3 years at Suizhou of OPT_+N_, OPT_–N_, FP, and CK were 12, 10, 9, and 8 t ha^–1^, respectively.

**TABLE 2:** Grain yield (GY) and yield components at Jingzhou.

Year (Y)	Treatment (T)	GY (t ha^–1^)	P (m^2^)	SP	GF (%)	GW (mg)	HI (%)
2017	CK	6.93 d	180 d	191.0 d	85.4 a	22.2 a	51.4 a
	FP	9.47 c	227 c	219.8 c	82.9 b	22.3 a	47.4 b
	OPT**_–_**_N_	10.07 b	233 b	230.1 b	80.7 c	21.8 b	47.9 b
	OPT_+N_	11.15 a	256 a	244.3 a	78.2 d	21.7 b	46.5 c
	***Mean***	***9.4***	***224***	***221***.***3***	***81***.***8***	***22***.***0***	***48***.***3***
2018	CK	7.01 d	221 c	191.3 c	84.5 a	20.9 b	46.8 b
	FP	9.29 c	245 b	218.7 b	82.6 bc	21.4 a	50.5 a
	OPT**_–_**_N_	10.10 b	258 ab	226.6 b	82.5 c	21.7 a	50.9 a
	OPT_+N_	11.22 a	277 a	241.9 a	82.4 ab	21.5 a	49.5 a
	***Mean***	***9***.***4***	***250***	***219***.***6***	***83***.***0***	***21***.***4***	***49***.***4***
2019	CK	6.27 d	190 c	196.8 d	81.1 a	20.2 b	45.8 b
	FP	7.57 c	230 b	241.1 c	75.2 c	20.4 ab	46.9 ab
	OPT**_–_**_N_	9.37 b	263 a	259.8 b	76.6 c	20.9 4 a	45.6 b
	OPT_+N_	10.97 a	271 a	272.1 a	79.3 b	20.4 ab	48.5 a
	***Mean***	***8***.***5***	***238***	***242***.***5***	***78***.***0***	***20***.***5***	***46***.***7***
2020	CK	6.16 d	168 d	246.7 c	79.6 a	19.3 c	47.3 a
	FP	8.17 c	216 c	263.2 bc	74.3 b	20.0 b	45.5 c
	OPT**_–_**_N_	9.11 b	231 b	276.4 ab	75.1 b	20.7 a	46.3 b
	OPT_+N_	10.85 a	275 a	306.7 a	74.3 b	20.6 a	45.7 c
	***Mean***	***8***.***6***	***222***	***273***.***3***	***75***.***8***	***20***.***1***	***46***.***2***
Analysis of variance	Year (Y)	0.97	19**	98.45**	22.37**	54.01**	9.51**
	Treatment (T)	147.73**	59**	13.25**	1.08	4.03*	0.04
	Y × T	16.27**	12**	4.15**	6.05**	10.15**	12.84**

P, panicles; SP, spikelets per panicle; GF, grain filling; GW, grain weight; HI, harvest index; ns, not significant. Lowercase letters within columns indicate significant differences at P < 0.05; *P < 0.05; **P < 0.01.

**TABLE 3:** Yield and yield components from 2017, 2018, and 2019 in Suizhou.

Year (Y)	Treatment (T)	GY (t ha^–1^)	P (m^2^)	SP	GF (%)	GW (mg)	HI (%)
2017	CK	8.14 d	177 d	201.5 d	88.2 a	22.8 a	51.3 a
	FP	9.36 c	231 c	226.9 c	86.7 b	22.5 b	48.4 b
	OPT**_–_**_N_	10.65 b	240 b	235.9 b	86.2 b	22.7 a	47.4 b
	OPT_+N_	12.23 a	270 a	260.3 a	84.5 c	22.5 b	46.3 c
	***Mean***	***10.7***	***238***	***206.8***	***86.4***	***22.6***	***48.4***
2018	CK	7.77 d	225 d	200.6 d	83.8 a	20.9 d	50.4 a
	FP	8.99 c	242 c	217.1 c	83.7 a	21.1 c	49.8 b
	OPT**_–_**_N_	10.00 b	255 b	226.0 b	83.3 a	21.6 b	47.8 c
	OPT_+N_	11.08 a	273 a	235.8 a	82.6 b	21.8 a	47.0 d
	***Mean***	***9.5***	***249***	***219.9***	***83.4***	***21.3***	***48.8***
2019	CK	7.70 d	225 d	235.7 c	78.2 a	21.8 a	47.3 c
	FP	9.63 c	263 c	242.6 bc	75.8 b	21.6 ab	47.9 b
	OPT**_–_**_N_	10.07 b	312 b	251.6 b	76.2 ab	21.4 ab	50.2 a
	OPT_+N_	11.60 a	330 a	265.5 a	76.3 ab	21.1 b	47.1 c
	***Mean***	***9.75***	***283***	***248.9***	***76.6***	***21.5***	***48.1***
Analysis of variance	Year (Y)	15.64**	7**	8.74**	378.68**	61.96**	0.42
	Treatment (T)	53.90**	15**	14.53**	0.41	0.12	5.28**
	Y × T	88.90**	24**	769**	3.03*	11.44**	14.29**

P, panicles; SP, spikelets per panicle; GF, grain-filling; GW, grain weight; HI, harvest index; ns, not significant. Different lowercase letters within columns indicate significant differences at P < 0.05; *P < 0.05; **P < 0.01.

Panicle number and spikelets per panicle were the highest under OPT_+N_, while there were significant differences across treatment systems at the two sites. Grain yields of treatments at both the sites were ranked in OPT_+N_ > OPT_–N_ > FP > CK. The average panicles number of OPT_+N_ was the highest (279), which was around 9 and 18% higher than that of OPT_–N_ and FP, respectively. Average spikelets per panicle of OPT_+N_ were 7 and 12% greater than that of OPT_–N_ and FP, respectively. Differences in grain filling rates and grain weight across the sites or between treatments were not significant.

### Leaf Area Index and Dry Matter Accumulation

A significant disparity was observed in LAI at the flowering stage (OPT_+N_ > OPT_–N_ > FP > CK; Tables 4, 5). OPT_+N_ had the highest mean LAI of 8, 17, and 32% higher than that of OPT_–N_ and FP, respectively. The aboveground total dry weight (TDW) of OPT_+N_ in Jingzhou and Suizhou at MA was 2,255 g m**^–^**^2^, which was around 9 and 19% greater than that of OPT_–N_ and FP, respectively, while OPT_–N_ was 10% greater than FP. Differences in harvest indices across the treatments and sites were not significant.

**TABLE 4:** Leaf area index (LAI) at flowering (FL), aboveground total dry weight (TDW), radiation use efficiency (RUE), and related parameters at maturity for four treatments at Jingzhou.

Year (Y)	Treatment (T)	LAI at FL	IR (MJ m^–2^)	IPAR (MJ m^–2^)	LIP (%)	TDW (g m^–2^)	RUE (g MJ^–1^)
2017	CK	3.49 c	1,820	487.7 d	78.4 d	1,153.5 d	2.37 d
	FP	6.45 b	1,836	639.0 c	81.5 c	1,580.0 c	2.47 c
	OPT**_–_**_N_	6.60 b	1,836	661.8 b	84.5 b	1,675.2 b	2.53 b
	OPT_+N_	7.08 a	1,847	722.3 a	87.4 a	1,876.6 a	2.60 a
	***Mean***	***5.9***	***1,835***	***627.7***	***83***	***1571.3***	***2.49***
2018	CK	3.71 c	1,753	723.7 d	68.5 d	1,516.0 d	2.09 c
	FP	5.90 b	1,755	788.1 c	76.8 c	2,013.8 c	2.56 b
	OPT**_–_**_N_	6.63 b	1,795	822.4 b	84.3 b	2,134.8 b	2.60 ab
	OPT_+N_	8.49 a	1,847	853.3 a	87.9 a	2,288.6 a	2.68 a
	***Mean***	***6.18***	***1,509***	***796.9***	***79.4***	***1,988.3***	***2.48***
2019	CK	2.52 d	1,943	554.3 c	51.1 d	1,201.4 d	2.17 c
	FP	5.52 c	1,962	743.7 b	75.2 c	2,039.0 c	2.74 b
	OPT**_–_**_N_	7.34 b	1,987	764.2 ab	82.8 b	2,155.5 b	2.82 a
	OPT_+N_	8.66 a	2,163	783.3 a	86.2 a	2,240.5 a	2.86 a
	***Mean***	***6.01***	***2,000***	***711.4***	***73.8***	***1,909.1***	***2.65***
2020	CK	2.72 c	1,630	533.1 d	59.0 d	1,193.2 d	2.24 c
	FP	4.79 b	1,716	650.2 c	77.6 c	1,700.0 c	2.61 b
	OPT**_–_**_N_	5.78 a	1,741	737.0 b	83.5 b	1,993.2 b	2.70 ab
	OPT_+N_	6.10 a	1,763	770.4 a	86.5 a	2,147.5 a	2.79 a
	***Mean***	***4.85***	***1,711***	***672.6***	***76.7***	***1,758.5***	***2.59***
Analysis of variance	Year (Y)	1.82*	–	8.48**	5.88*	3.22*	7.85**
	Treatment (T)	12.33**	–	20.55**	12.54**	51.21**	61.77**
	Y × T	21.01*	–	55.88**	28.20**	11.24**	32.34**

IR, total solar radiation; IPAR, intercepted photosynthetically active radiation; LIP, light interception percentage; ns, not significant. Different lowercase letters within columns indicate significant differences at P < 0.05; *P < 0.05; **P < 0.01.

**TABLE 5:** Leaf area index at FL, aboveground TDW, RUE, and related parameters at maturity for four treatments at Suizhou.

Year (Y)	Treatment (T)	LAI at FL	IR (MJ m^–2^)	IPAR (MJ m^–2^)	LIP (%)	TDW (g m^–2^)	RUE (g MJ^–1^)
2017	CK	3.76 c	1,982	722 c	72 c	1,629 d	2.2 c
	FP	6.31 b	2,062	879 b	85 b	2,043 c	2.3 c
	OPT**_–_**_N_	7 a	2,104	929 a	88 a	2,261 c	2.4 b
	OPT_+N_	7.63 a	2,119	948 a	89 a	2,461 a	2.6 a
	***Mean***	***6.2***	***2,067***	***872***	***84***	***2,099***	***2.4***
2018	CK	3.82 c	2,009	725 c	74 c	1,538 c	2.1 c
	FP	6.88 b	2,131	819 b	84 b	2,073 b	2.5 b
	OPT**_–_**_N_	7.14 b	2,036	848 ab	85 b	2,133 b	2.5 b
	OPT_+N_	8.14 a	2,064.1	922.8 a	89.5 a	2,421.1 a	2.62 a
	***Mean***	***6.5***	***2,060***	***829***	***86***	***2,041***	***2.4***
2019	CK	6.68 d	1,979	731 c	77 c	1,441 d	1.9 c
	FP	7.29 c	2,036	856 b	84 b	1,823 c	2.1 c
	OPT**_–_**_N_	8.29 b	2,131	877 b	85 b	2,174.2 b	2.4 b
	OPT_+N_	10.93 a	2,175	899 a	90 a	2,348 a	2.6 a
	***Mean***	***8.3***	***2,080***	***841***	***84***	***1,946***	***2.3***
Analysis of variance	Year (Y)	11.81*	–	0	0	0	3.8*
	Treatment (T)	2.39**	–	120**	25**	250**	24.1**
	Y × T	15.27*	–	5**	9**	4**	20.6**

IR, total solar radiation; IPAR, intercepted photosynthetically active radiation; LIP, light interception percentage; ns, not significant. Different lowercase letters within columns indicate significant differences at P < 0.05; *P < 0.05; **P < 0.01.

### Radiation Use Efficiency

The average IPAR of OPT_+N_ was 843 MJ m**^–^**^2^, 5 and 10% higher than that of OPT_–N_ and FP, respectively (Tables 4, 5). The average light interception percentage (LIP) of OPT_+N_ was 88, 4, and 9% higher than that of OPT_–N_ and FP, respectively. The average RUE of OPT_+N_ was 2.7 g MJ**^–^**^1^, 5 and 9% greater than the RUE of OPT_–N_ and FP, respectively, while the RUE of OPT_–N_ was 4% higher than that of FP.

### Nitrogen Use Efficiency

The agronomic use efficiency of nitrogen fertilizer (AE_N_) differed significantly across treatments and trends were mostly consistent across years and sites, with OPT_+N_ > OPT_–N_ > FP (Table 6). The average AE_N_ of OPT_+N_ was 17 kg grain kg**^–^**^1^ N, 9 and 68% higher than OPT_–N_ and FP, respectively. The RE_N_ in Jingzhou and Suizhou sites showed generally steady trends.

**TABLE 6:** Agronomic efficiency of applied N (AE_N_), partial factor productivity of applied N (PFP_N_), and crop recovery efficiency of applied N (RE_N_) in Jingzhou and Suizhou.

Year (Y)	Treatment (T)	AE_N_ (kg/kg)		PFP_N_ (kg/kg)		RE_N_ (%)	
		Jingzhou	Suizhou	Jingzhou	Suizhou	Jingzhou	Suizhou
2017	FP	12.12 c	18.88 b	45.11 b	59.56 a	50.98 a	66.09 a
	OPT**_–_**_N_	16.11 a	22.95 a	51.64 a	60.51 a	57.46 b	63.39 b
	OPT_+N_	15.64 a	22.76 a	41.30 c	51.48 b	39.60 c	22.65 c
	***Mean***	***14***.***62***	***21***.***53***	***46***.***02***	***57***.***18***	***49***.***35***	***50***.***71***
2018	FP	10.83 c	4.89 c	41.54 c	37.37 c	31.18 c	59.45 c
	OPT**_–_**_N_	15.81 b	11.98 b	51.79 a	50.72 a	54.19 b	90.89 b
	OPT_+N_	17.56 a	15.16 a	44.23 b	45.29 b	66.63 a	95.22 a
	***Mean***	***14***.***73***	***10***.***68***	***45***.***85***	***44***.***46***	***50***.***67***	***81***.***85***
2019	FP	9.19 c	4.90 c	36.03 c	35.90 c	34.24 c	61.23 c
	OPT**_–_**_N_	15.90 b	10.66 b	48.03 a	47.64 a	51.16 b	109.13 b
	OPT_+N_	17.41 a	12.29 a	40.62 b	41.05 b	65.48 a	123.14 a
	***Mean***	***14***.***17***	***9***.***28***	***41***.***56***	***41***.***53***	***50***.***29***	***97***.***83***
2020	FP	9.56 c	–	38.89 c	–	29.15 c	–
	OPT**_–_**_N_	15.11 b	–	46.70 a	–	41.86 b	–
	OPT_+N_	17.38 a	–	40.20 b	–	48.73 a	–
	***Mean***	***14***.***02***	**–**	***41***.***93***	**–**	***33***.***91***	**–**
Analysis of variance	Year (Y)	0.25	140.42**	15.03**	268.94**	2.33	8.88**
	Treatment (T)	55.36	7.52**	81.00**	16.13**	11.02	2.69
	Y × T	12.83**	15.26**	24.02**	14.22**	24.13**	128.07**

Different lowercase letters within columns indicate significant differences at P < 0.05; *P < 0.05; **P < 0.01. ns, not significant.

### Correlation Analysis of Grain Yield and Yield Components

**FIGURE 1:**
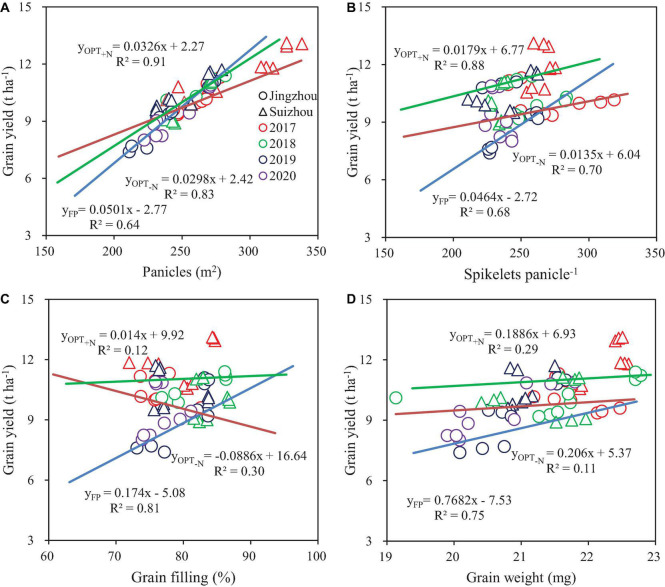
Relationships between grain yield and panicle number **(A)**, spikelets per panicle **(B)**, grain filling **(C)**, and grain weight **(D)**. Colors and point symbols represent different years and sites, respectively.

Grain yield was positively correlated with panicle number and spikelets per panicle within treatments and within sites (Figure 1). Panicle number and spikelets per panicle of OPT_+N_ were tightly coupled with yield, with *R*^2^ of 0.91 and 0.88, respectively. Relationships between grain yield and grain filling or grain weight of OPT_+N_ were not significant. Panicle number and spikelets per panicle of OPT_–N_ were correlated with yield, with *R*^2^ values of 0.83 and 0.70, respectively.

### Radiation Interception, Radiation Use Efficiency, and Correlation With Dry Matter and Yield

**FIGURE 2:**
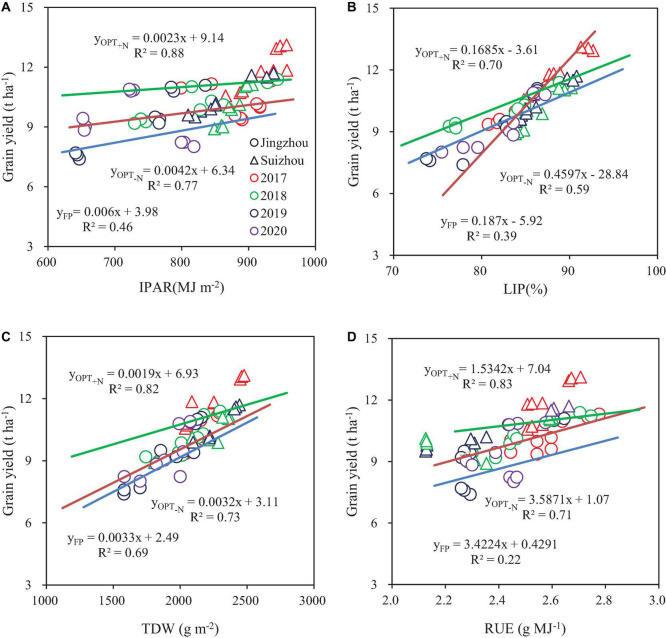
Relationship between grain yield and intercepted photosynthetically active radiation (IPAR) **(A)**, light interception percentage (LIP) **(B)**, aboveground total dry weight (TDW) **(C)**, and radiation use efficiency (RUE) **(D)**. Colors and point symbols represent different years and sites, respectively.

A significant correlation was observed between each of LIP, IPAR, TDW, and RUE with yield (Figure 2). IPAR had the highest correlation with yield under OPT_+N_ and OPT_–N_, with *R*^2^ values of 0.88 and 0.77, respectively. The *R*^2^ values of LIP, TDW, and RUE with yield were 0.70, 0.82, and 0.83 under OPT_+N_, respectively; the R^2^ values of LIP, TDW, and RUE with yield were 0.59, 0.73, and 0.71 under OPT_–N_, respectively.

## Discussion

Higher sink potential is a prerequisite for achieving higher rice yields (Huang et al., 2021). In the 1990s, medium-spike varieties (about 200 spikelets per panicle) were widely planted across China, while the large-spike varieties (more than 250 spikelets per panicle) were planted more sparingly (Ma and Yuan, 2015; Yuan, 2017). By 1996, Liangyoupeijiu met the criteria for defining super rice cultivars, having a strong source and sink and total spikelets of above 4.5 × 10^8^ ha^–1^; recently, the sink potential of genotypes Y-liangyou 1 and Y-liangyou 2 have reached 5.2 × 10^8^ spikelets ha^–1^, while that for Y-liangyou 900 and Xiangliangyou 900 have reached 6.0–7.5 × 10^8^ spikelets ha^–1^ (Li et al., 2009; Yuan, 2017). In this study, the average sink potential of the super rice variety YLY900 attained 7.2 × 10^8^ and 7.4 × 10^8^ spikelets ha^–1^ under OPT_+N_. Average yields were 11 t ha^–1^ in Jingzhou and 12 t ha^–1^ in Suizhou. The OPT_+N_ had higher N fertilizer application (270 kg N ha^–1^) and moderate dense planting (Jingzhou: 21 plants m^–2^; Suizhou: 30 plants m^–2^), which correspondingly expanded sink potential by improving panicle number and spikelets per panicle. Meanwhile, the OPT_+N_ improved N translocation patterns, which postponed N application to late season phenology, delayed leaf senescence, extended the filling period, and promoted the formation of high sink potential. This finding is similar to previous studies, which have shown that practices enabling extension of crop green area duration tend to raise yields (Harrison et al., 2012a,b). As a consequence of insufficient transplanting density (Jingzhou: 19 plants m^–2^; Suizhou: 25 plants m^–2^), panicle numbers of FP enabling potential yields were not realized. The results showed that an increase in N application (within reasonable levels) could increase the dry matter accumulation. Farmers in this region typically focused on the application of fertilizers at transplanting and early tillering stages but neglected N applications at PI and topdressing stages. Such management practices severely constrained potential yields of large-spike super rice genotypes because plant N demand in the later stage has not been met. Further, excessive N application in early crop life as well as improper N management in later stages could result in early leaf senescence and poor grain filling. We suggest that N fertilizer distributed and moderately dense planting should improve N use efficiency in super hybrid rice.

Grain yield was determined by biomass and harvest index. Thus, grain yield could be increased by the improvement of biomass production, harvest indices, or both (Battaglia et al., 2018; Thomason and Battaglia, 2020). However, previous studies have revealed that the yield of hybrid rice depends mainly on biomass, but there is little scope for increasing rice yield by improving the harvest index (Yang et al., 2009; Lu et al., 2021). In this study, harvest index differences across treatments were small, while variation in dry matter accumulation across treatments was significant, as the amount of dry matter accumulated after flowering accounts for 70–80% during the whole rice growing season (Venkateswarlu and Visperas, 1987). Maintaining adequate LAI through grain filling could ensure sufficient dry matter accumulation after flowering and, then, higher grain yields could be achieved (Huang et al., 2013). In this study, the TDW of OPT_+N_ was increased by 9 and 19% compared with OPT_–N_ and FP. The LAI of OPT_+N_ (8.2) at flowering was significantly higher than that of OPT_–N_ (7.0) and FP (6.2). It is worth noting that moderate dense planting and seasonal N fertilizer distribution management increased LAI, which improved light interception and dry matter accumulation (Saito et al., 2010). Postponing N application is conducive to a higher photosynthetic capacity of leaves in the grain filling stage as well as greater radiation interception (Yang and Zhang, 2010). Higher LAI of OPT_+N_ promoted accumulation of dry matter and more biomass was the main reason for high yield in this study.

Crop RUE is defined as the volume of biomass accumulated per unit solar radiation intercepted (Meek et al., 1984; Sinclair and Muchow, 1999). With morphological and intersubspecific heterosis improvement, significant yield advancement has occurred in super hybrid rice in the past few decades in China (Yuan, 2017). Further yield potential improvement mainly depends on greater RUE. Super hybrid rice populations have higher RUE and IPAR, resulting in higher biomass and yield (Zhang et al., 2009; Liu et al., 2019b). It has been proposed that the yield potential of hybrid rice varieties and RUE could further be increased at high N levels (Liu et al., 2019a). We found that the RUE of OPT_+*N*_ was increased by 5 and 9% compared with OPT**_–N_** and FP, respectively, and that equal seasonal N application with moderate dense planting improves light interception and RUE. Optimization of nitrogen retranslocation patterns could delay the senescence of leaves and improve radiation interception during the late grain filling stage. Our results showed that the yield of OPT_+N_ was clearly correlated with IPAR, IP, and RUE, with ***R*****^2^** values of 0.88, 0.70, and 0.83, respectively. This study suggested that increasing IP at midtillering and IPAR at the grain filling stage significantly improved RUE (Chen et al., 2019; Lu et al., 2020). These findings demonstrate that optimizing cultivation and nutrient management considerably increases the RUE of super hybrid rice and emphasizing appropriate practice is favorable to the environment.

Nitrogen fertilization is of great importance for rice growth and yield formation. It has been demonstrated that N application has a significant effect on the yield of super hybrid rice and appropriate seasonal management of N applications could improve TDW, yield, and NUE of rice (Wang et al., 2001). Super hybrid rice could achieve higher yield at both the low and high N levels, but the physiological and yield advantages were more favorable under high N conditions (Huang et al., 2016). The total N uptake per unit area of ultra-high yielding rice was high and the agronomic and partial productivity of N fertilizer was higher (Peng et al., 2002). In this study, the average AE_N_ of OPT_+N_ and OPT_–N_ was 68 and 54% higher than FP, respectively. The NUE of OPT_+N_ and OPT_–N_ improved because (1) improved management for OPT_+N_ and OPT_–N_ (i.e., later season N applications and moderate dense planting) increased panicle number and spikelets per panicle and raised NUE and yield and (2) in the early stage of rice crop life cycle, seedlings were generally too weak to fully utilize N fertilizer, so that superfluous N fertilizer at this stage increased the number of invalid tillers, resulting in poor yield and NUE (Liu et al., 2009). Meanwhile, we showed that alternative wet and dry cycles during the critical rice fertility period improved NUE. Our results also suggested that more equal seasonal N distribution, adoption of alternative wet-dry cyclical management, and moderate planting density could synergistically improve resource use efficiency and yields.

## Conclusion

Here, we investigated the TDW, RUE, NUE, and yield components of super hybrid rice using several treatment regimens over two sites and 4 years. We discovered that (1) appropriate increases to applied nitrogen fertilizer increased panicle number and spikelets per panicle, which expanded sink potential and raised grain yield, (2) equi-seasonal N fertilizer application in concert with moderate density planting were instrumental to enhancing dry matter accumulation and yields in super hybrid rice, (3) higher LAI, IP, and IPAR of super hybrid rice underpinned the higher RUE observed for these hybrids, and (4) super hybrid rice has compared with historical and non-hybrid genotypes, super hybrid rice generally has greater agronomic efficiency and crop recovery of N, leading to higher nitrogen-use efficiencies and higher radiation-use efficiency. Collectively, these factors are conducive to higher yields.

## Data Availability Statement

The original contributions presented in the study are included in the article/Supplementary Material, further inquiries can be directed to the corresponding author/s.

## Author Contributions

YZ designed the experiments and revised the manuscript. JD, JY, and XX investigated the traits. JD analyzed the data. JD and MH wrote the manuscript. MH, KL, SF, LH, and XT aided with conceptualization, scientific rigor, and manuscript editing. All authors contributed to the article and approved the submitted version.

## Conflict of Interest

The authors declare that the research was conducted in the absence of any commercial or financial relationships that could be construed as a potential conflict of interest.

## Publisher’s Note

All claims expressed in this article are solely those of the authors and do not necessarily represent those of their affiliated organizations, or those of the publisher, the editors and the reviewers. Any product that may be evaluated in this article, or claim that may be made by its manufacturer, is not guaranteed or endorsed by the publisher.
