# Supplementary material for: Integrated Crop Management Practices Improve Grain Yield and Resource Use Efficiency of Super Hybrid Rice
Source: Front Plant Sci. 2022 Mar 30;13:851562. doi: 10.3389/fpls.2022.851562 (PMC9007698; doi:10.3389/fpls.2022.851562)
Supplement: Supplementary file 1 [file Data_Sheet_1.docx]

**S1.** Daily maximum temperature, minimum temperature, and solar radiation from transplanting to maturity in 2017 (A), 2018 (B), 2019 (C) and 2020 (D) in Jingzhou, Hubei Province, China.

**S2.** Daily maximum temperature, minimum temperature, and solar radiation from transplanting to maturity in 2017(A), 2018(B), and 2019 (C) in Suizhou, Hubei Province, China.
